# Educational Method to Promote Recognition, Diagnosis, and Treatment of Eating Disorders: A Cluster-Randomized Study of the Effects of Retrieval Practice Amongst Medical Trainees

**DOI:** 10.7759/cureus.71552

**Published:** 2024-10-15

**Authors:** Maria D Ash, Cynthia Holland-Hall, Kenneth Jackson, Guy N Brock, Andrea E Bonny

**Affiliations:** 1 Adolescent Medicine, Nationwide Children’s Hospital, Ohio State University College of Medicine, Columbus, USA; 2 Biomedical Informatics, Ohio State University College of Medicine, Columbus, USA; 3 Center for Biostatistics, Ohio State University Wexner Medical Center, Columbus, USA; 4 Biostatistics Resource at NCH (BRANCH), Nationwide Children’s Hospital, Ohio State University, Columbus, USA; 5 Pediatrics, Nationwide Children’s Hospital, Ohio State University, Columbus, USA

**Keywords:** adolescent medicine, eating disorders (eds), medical education research, retrieval practice, teaching and training residents and medical students

## Abstract

Background

Eating disorder (ED) education during medical training is lacking. Few medical trainees feel comfortable managing EDs, and an alarming 78% of healthcare providers report feeling insecure in treating EDs. Recognizing EDs early is crucial as the standardized mortality ratio for patients with anorexia nervosa is more than five times higher than that of the general population. Retrieval practice is a powerful tool in producing meaningful learning of complex concepts in education. We investigated the effectiveness of retrieval practice in ED education among medical trainees to improve recognition, diagnosis, and treatment of EDs among adolescent patients.

Methods

This exploratory, prospective, cluster-randomized trial enrolled residents and medical students over 14, four-week blocks. Participants were randomized by block to either the conventional lecture-based format (control group) or the retrieval-based educational format (intervention group). The control group received case-based lectures. The intervention group received education via 21 case-based quiz questions over the block with immediate feedback. Groups completed nine-item, multiple choice pre (T1)- and post (T2)-rotation knowledge tests, covering recognizing, diagnosing, and treating EDs. All participants also completed pre- and post-rotation surveys designed to measure self-perceived comfort, confidence in training, knowledge, and skills on the topic of EDs.

Results

The study’s primary outcome was the difference between T1 and T2 scores between study groups. The intervention group showed greater improvement from T1 to T2 (5.8 to 7.4, respectively) than the control group (5.1 to 6.0, respectively). The difference between mean T1 and T2 scores in the control group versus the intervention group was significant (p=0.020). Despite the control group reporting improvements in confidence regarding training on EDs, this increased confidence was inversely correlated with scores on T2 (r=-0.502, p=0.011).

Conclusions

Trainees benefit from retrieval practice to improve knowledge acquisition regarding EDs. Standard lectures may confer false confidence to learners, which may not accurately align with actual knowledge acquisition.

## Introduction

Eating disorder (ED) education has long lacked appropriate instruction during medical training [1,2]. A persistent eating disturbance that results in altered food consumption characterizes EDs and significantly impairs physical health and psychosocial functioning [3]. The lifetime prevalence of any ED among a community cohort of adolescents is 5.7% among women and 1.2% among men [4]. The standardized mortality ratio for anorexia nervosa (AN) is more than five times higher than that in the general population matched for age and sex [5]. Complications of AN include derangement in hematologic parameters, impairment of bone mass accrual, and primary or secondary menstruation suppression [6,7]. Cardiovascular changes noted in patients with AN include bradycardia, hypotension, orthostatic vital sign changes, QT interval prolongation, myocardial atrophy, and pericardial effusion [7,8].

Given the morbidity and mortality associated with EDs, early recognition and intervention are crucial [9]. A long duration substantially raises the risk of mortality [9,10]. Primary care providers are often the first point of contact with patients struggling with EDs, yet these providers lack knowledge of specific characteristics of these illnesses such as enlarged parotid glands and delayed gastric emptying, both of which could be presenting symptoms of patients [11]. An alarming 78.0% of providers were unsure how to treat patients with an ED, and 92.0% felt they had missed an ED diagnosis [12]. Medical trainees lack basic knowledge and training in EDs, including diagnostic criteria, prevalence rates, and effective treatment. Only a minority of trainees, 11.4%, felt comfortable managing patients with EDs [2]. Medical trainees require educational intervention to improve the recognition, diagnosis, and treatment of EDs.

Under the umbrella of cognitive learning theory, retrieval practice is a well-studied concept. Retrieval practice, also known as “testing,” “testing effect,” or “test-enhanced learning,” is a learning strategy that demands effortful recall to consolidate memories into a connected fluid representation in the brain and to strengthen and multiply the neural routes for later knowledge recall [13]. Retrieval practice is a successful and powerful tool in promoting and producing the learning of complex concepts found in science education and professional development [14,15]. The use of multiple-choice questions can construct knowledge and significantly improve and enhance meaningful learning [15-17]. Retrieval practice has been used successfully in secondary classrooms, post-secondary pharmacy and anatomy classes, and in continuing professional development for physicians [14,18-23]. Cognitive learning theory, in particular, retrieval practice, was chosen as the foundation for this study due to the ease with which this theory can be applied in clinical teaching and its implications for long-term retention and hoped-for translation into medical knowledge and practice [24].

Feedback is an essential element of effective retrieval practice [25-27]. The timing of feedback has been a nuanced topic. Immediate, timely feedback is appropriate in some situations such as during task acquisition, and less superior in other cases such as in simulation-based education [28-30]. Importantly, as students make errors in test-like events during instruction, feedback has a more substantial effect on the later retrieval of correct information [31]. Trainees in higher-level medical courses who receive detailed feedback after retrieval demonstrate increased knowledge and improved performance [32,33].

This exploratory study’s primary objective was to assess the impact of an educational method that utilizes retrieval practice with immediate feedback compared to the standard lecture format on medical trainee knowledge acquisition regarding proper recognition, diagnosis, and treatment of EDs. We hypothesized that medical trainees receiving retrieval-based learning would acquire more knowledge than the control group as measured by a nine-item knowledge test measuring correct ED recognition, diagnosis, and treatment administered at the beginning and end of the four-week adolescent medicine rotation.

A secondary aim was to explore retrieval practice’s efficacy in improving medical trainee self-perceived comfort, knowledge, and skills regarding managing EDs, as well as confidence in their training. We hypothesized that medical trainees receiving retrieval-based learning would have a more pronounced improvement in self-perceived comfort, confidence in training, knowledge, and skills than the control group as assessed by a five-point Likert scale on a survey administered at the beginning and end of the four-week adolescent medicine rotation.

## Materials and methods

Participants

The Accreditation Council for Graduate Medical Education (ACGME) requires that all residents complete a one-month rotation in adolescent medicine as a pediatric residency program requirement or combined internal medicine-pediatrics residency program requirement [34,35]. Fourth-year medical students can elect to complete a four-week elective rotation in adolescent medicine. Medical trainees were prospectively invited to participate in a cluster-randomized educational pilot study during their adolescent medicine rotation at a large pediatric health and research institution in the Midwestern United States during the academic years 2018-2019 and 2019-2020. These participants were recruited over 14, four-week blocks. Each block contained one to five learners. Blocks were not enrolled consecutively due to holiday block rotations or other conflicts. Learners on two-week split blocks (e.g., 2-Week Adolescent Medicine/2-Week Developmental-Behavioral Pediatrics block) were welcome to participate in educational activities but were excluded from the study. Consented participants who completed the study included fourth-year medical students (MS4) (15.2%), first-year residents (post-graduate year, PGY1) (19.6%), second-year residents (PGY2) (4.3%), third-year residents (PGY3) (43.5%), and fourth-year residents (PGY4) (17.4%). The institutional review board provided exempt status for this study under 45 CFR 46.101(b)(1). 

Power calculation

Detectable effect sizes (80% power, alpha = 0.05) were determined for the cluster-randomized design with eight clusters per arm, an average cluster size of three to four, and an intraclass correlation ranging between 0.01 and 0.05. The design has 80% power to detect an effect size of roughly 0.85, with three residents per cluster (n=48) and 0.75 with four residents per cluster (n=64). Recruitment ended prior to reaching eight clusters per arm secondary to external time constraints and the COVID-19 pandemic. PASS2020 (Power Analysis and Sample Size, version 16) software (NCSS Statistical Software, NCSS, LLC, Kaysville, UT) calculated power.

Design and randomization

Learners were provided with a study description before verbally consenting. The medical trainees were randomly assigned using concealed allocation by block to either the conventional lecture-based format (control group) or the retrieval-based educational format (intervention group) using a block randomization scheme with block sizes of two and four. Groups were blinded to intervention and control procedures. Both groups completed nine-item, multiple-choice, pre (T1)- and post (T2)-rotation knowledge tests on the first Wednesday (week 1) and last Wednesday (week 4) of the rotation. Additionally, all participants completed a pre (S1)- and post (S2)-rotation attitude survey, which was designed to measure self-perceived comfort, confidence in training, knowledge, and skills in managing EDs.

Physician learning begins with an individual becoming aware of a problem or challenge [36]. Both the control and intervention groups participated in a case-based interactive learning activity during week 1. This introduction to the block allowed trainees the opportunity to become aware of their specific knowledge gaps in EDs. Because the participants were coming onto the rotation at different points in their training, the activity also served to provide all trainees with a comparable starting point before beginning their respective learning activities for the block. The Society for Adolescent Health and Medicine website section on the Eating Disorders & Overweight/Obesity education page provided this case-based activity [37].

**Figure 1:**
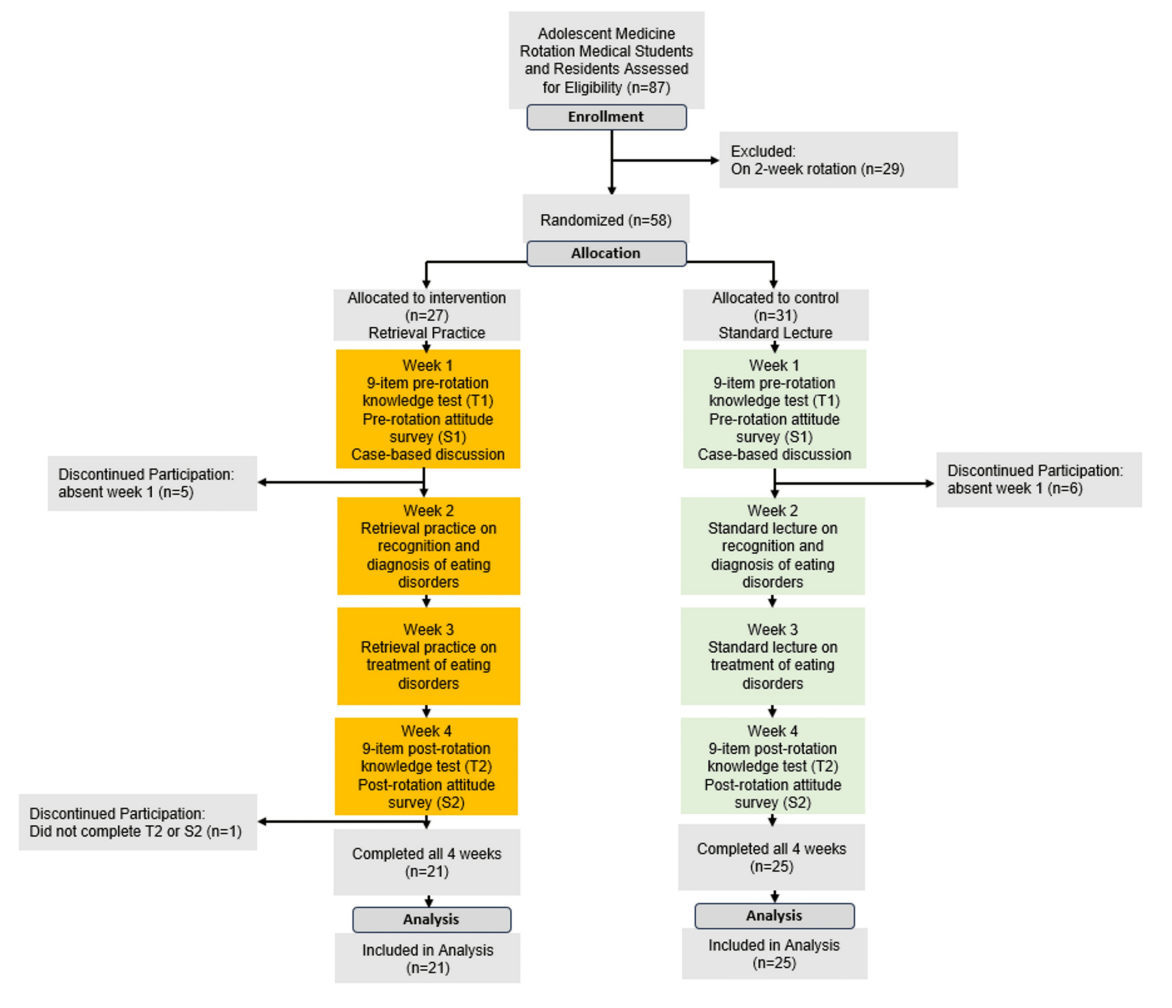
CONSORT flow chart of the randomized controlled trial. Students were defined as discontinuing participation if they were absent or did not complete outcome measures. Attrition was similar between groups. CONSORT: Consolidated Standards of Reporting Trials

The control group received lectures on ED recognition and diagnosis based on data supplements released in conjunction with an article published in Pediatrics in Review, entitled “Eating Disorders” on the second Wednesday of the rotation (week 2) [38,39]. During the third Wednesday of the rotation (week 3), the control group received education on treating EDs using the lecture format created by the same literature [38,39]. The intervention group received education via 21 case-based quiz questions over the block with immediate feedback. Week 2 of the rotation covered recognizing and diagnosing EDs, and week 3 contained treating EDs. The same literature was used in creating this material to ensure each group was exposed to the same information in the experimental protocol (Figure 1).

Outcome measures

Pre (T1)- and Post (T2)-Rotation Knowledge Test

The study’s primary outcome was the difference between the pre (T1)- and post (T2)-rotation nine-item knowledge test scores between study groups. The nine-item knowledge test contained three questions each on ED recognition, diagnosis, and treatment. The test was multiple choice with a “best answer.” The American Board of Pediatrics objectives guided developing questions in their various content outlines and content specification resources [40,41]. Medical trainees tested pilot questions from July to October 2018 to demonstrate discriminatory capacity. In addition, three versions of each query were developed to randomly assign each medical trainee a unique rendering of each question at each assessment point to decrease intraclass correlation.

Pre (S1)- and Post (S2)-Rotation Attitude Survey

A secondary outcome was the difference between study groups in self-perceived comfort, confidence in training, knowledge, and skills from the beginning to the end of the rotation. The survey was adapted from previous studies and consisted of various items, including the Likert scale and dichotomous items [11,12]. The pre-rotation survey included additional questions to obtain demographic information.

Statistical analysis

Learners who completed the entire study (no absences, completed outcome measures) were included in the analysis. Attrition was similar between groups. Descriptive analyses, including counts, percentages, means and standard deviations, were performed to illustrate distributions of key variables. Linear models were used to compare the change in the following outcome measures from the beginning of the rotation to the end of the rotation between groups: knowledge totals on T1-T2, skills items on S1-S2, and knowledge items on S1 and S2. For each, the second value was considered a response with the baseline value and group assignment as covariates. Due to fitting issues, the block was not included as a random effect for any model, except the knowledge totals on T1-T2. Estimates and 95% confidence intervals (CI) were reported along with p-values for these tests. Concerning the change in knowledge totals on T1-T2, an individual p-value of <0.05 was considered statistically significant. Spearman’s rank correlation quantified the degree to which differences in reported comfort managing patients with EDs on S2 corresponded to differences in T2 score (without assuming a particular relationship); associated t-tests were run to enable inferences regarding those values. Using a Bonferroni correction across five tests overall, an individual p-value of <0.01 was deemed statistically significant for that specific instance. All statistical analyses were conducted using SAS 9.4 (SAS Institute, Cary, NC).

## Results

Demographics

Of the 46 enrolled medical trainees who completed the study, the control group included 25 participants and the intervention group included 21 participants (Table 1). One resident in each group did not disclose race; one participant in the control group did not divulge age. The majority of learners in both groups were white, pediatric trainees. PGY3 residents outnumbered other training years in both groups. The participant’s ages were similar in each group. The control group was comprised of more residents in their PGY1 year of training than the intervention group, which included slightly more PGY4 residents in a combined internal medicine-pediatrics residency program. Regarding formal education on EDs, 36.9% (17/46) of participants reported receiving “0” hours during their medical/residency training, while about 58.7% (27/46) reported receiving one to four hours of formal ED education. In terms of experience treating patients with EDs, 67.4% of learners reported treating five or fewer patients throughout their training.

**Table 1:** Demographics of participating students. †One participant in each group was missing a value identifying race; ‡ One participant in the control group was missing a value identifying age MS4 = Medical Student, 4th year; PGY = Post-Graduate Year

Characteristic	Control, n (%)	Intervention, n (%)
Mean Age, y	28.5^‡^	30.4
Race^†^		
White	21 (84.0)	14 (66.7)
Non-White	3 (12.0)	6 (28.6)
Training Year		
MS4	4 (16.0)	3 (14.3)
PGY1	7 (28.0)	2 (9.5)
PGY2	2 (8.0)	0 (0.0)
PGY3	10 (40.0)	10 (47.6)
PGY4	2 (8.0)	6 (28.6)
Residency Type		
N/A (medical student)	4 (16.0)	3 (14.3)
Family Medicine	3 (12.0)	2 (9.5)
Internal Medicine/Pediatrics	2 (8.0)	5 (23.8)
Pediatrics	16 (64.0)	11 (52.4)

Knowledge test results

**Figure 2:**
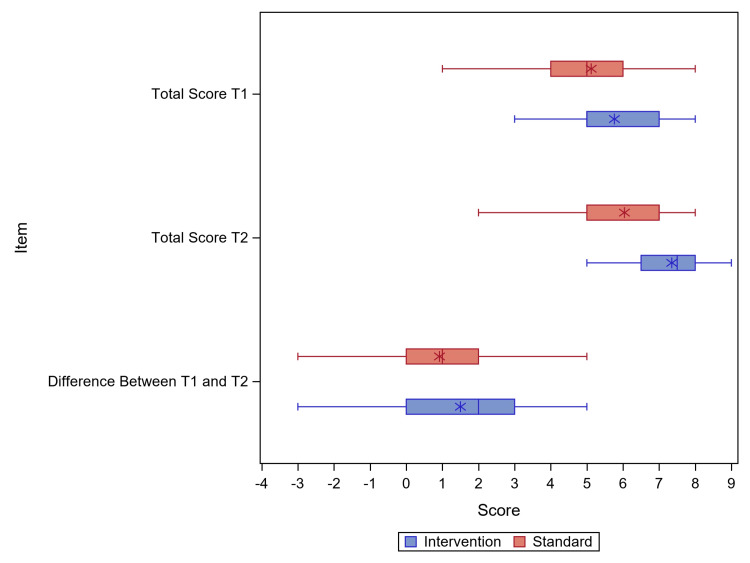
Change in the mean score and difference between pre- and post-rotation knowledge test scores. * represents mean; | represents mode

All knowledge tests had a maximum possible score of 9.0. The control group’s mean score was 5.1 at T1, and 6.0 at T2. The intervention group’s mean score was 5.8 at T1, and 7.4 at T2 (Figure 2).

The difference between the mean scores in the control group versus the intervention group was significant based on a linear model (beta=1.308; 95% CI: 0.217, 2.398; p=0.020; ICC=0.17). The means and standard deviations for each group are listed in Table 2.

**Table 2:** Mean knowledge test scores.

	Control (n=25)	Intervention (n=21)
Total Score T1, mean +/- SD	5.1 +/- 1.6	5.8 +/- 1.5
Total Score T2, mean +/- SD	6.0 +/- 1.6	7.4 +/- 1.3
Difference Between T1 and T2, mean +/- SD*	0.9 +/- 2.0	1.2 +/- 2.3

Survey results

Participants were asked, “How comfortable are you in managing patients with eating disorders?” In the control group, 12 of 25 (48.0%) showed greater comfort managing patients with EDs by the end of the rotation; 11 of 25 (44.0%) did not have a change in their comfort level; and 2 of 25 (8.0%) showed worsened comfort in managing patients with EDs. In the intervention group, 13 of 20 (65.0%) trainees showed greater comfort managing patients with EDs; six of 20 (30.0%) did not have a change in their comfort level; and one of 20 (5.0%) showed worsened comfort.

Trainees were assessed on confidence in their training pertaining to EDs. Participants were asked whether they felt well-trained to recognize, diagnose, and treat patients with EDs. Control group responses had a negative correlation between the reported level of confidence regarding training on EDs on S2 and performance on T2 (r=-0.502, p=0.011). No such correlation was found for the intervention group (r=0.089, p=0.709).

**Figure 3:**
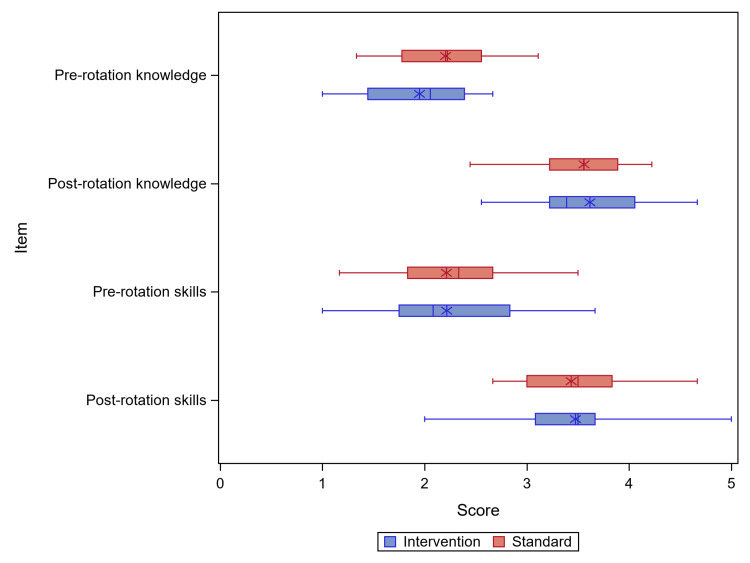
Change in the mean score of self-perceived knowledge and skills. * represents mean; | represents mode

Both the control and intervention groups showed improvement in self-perceived knowledge and skills (Figure 3). The values were similar across groups, with no statistically significant difference between groups in mean change in scores from S1 to S2 self-perceived knowledge scores (beta=0.069; 95% CI: -0.268, 0.407; p=0.680) or self-perceived skills scores (beta=-0.009; 95% CI: -0.377, 0.360; p=0.962).

Trainees provided feedback on S2 regarding their satisfaction with the educational format. In the control group, 17 of 25 (68.0%) respondents were extremely satisfied with their learning experience, six of 25 (24.0%) were somewhat satisfied, and two of 25 (8.0%) were extremely unsatisfied. In the intervention group, seven of 20 (35.0%) respondents were extremely satisfied with their learning experience, nine of 20 (45.0%) were somewhat satisfied, 1 of 20 (5.0%) was somewhat unsatisfied, and three of 20 (15.0%) were extremely unsatisfied.

## Discussion

The current study is the first published using a prospective cluster-randomized trial design to pilot a potential method for educating residents and medical students on ED recognition, diagnosis, and treatment. In education specific to EDs, few trainees receive adequate lecture time on the topic, and even fewer can gain experience by directly treating patients with EDs [1,2]. Our results, unfortunately, confirmed this finding, with almost all trainees reporting receiving less than four hours of formal education and nearly 70% having treated only five patients or less up to that point in their training.

A common theme in medical education reform is the need for research on best practices in medical education [42]. Research supports active learning, which is learning that introduces activity and promotes student engagement [43]. ED education has not met students’ needs in medical education and reflects the lack of preparedness in primary care physicians and other healthcare providers [2,11,12,44-48]. Our study demonstrates that retrieval practice may be a viable method to educate medical trainees on EDs. The difference between mean scores in the control group versus the intervention group was significant, suggesting a possible benefit to using retrieval practice in medical trainees’ education on EDs. A possible explanation for the improved performance of the intervention group is that evaluating one’s own knowledge base through retrieval practice may result in a more engaged learner. Immediate feedback given during instruction in the intervention group further strengthened knowledge by correcting previously misunderstood information [32,33].

The control group began the rotation with more participants reporting comfort managing patients with EDs than the intervention group. By the end of the rotation, post-rotation survey scores showed more movement towards increasing comfort in the intervention group as compared to the control group. This increase is likely because retrieval practice recalibrates one’s understanding of what they do and do not know [13]. This recalibration allows for increased comfort in one’s knowledge base. 

While the control group showed a shift towards increased confidence regarding training on EDs during the rotation, our data show a negative correlation between displaying increased confidence in training on EDs and final test scores in the control group; as confidence increased, knowledge test scores decreased. This finding may stem from the control group lacking the opportunity to test and refine their knowledge, falsely inflating their abilities, leading them to confuse their familiarity with presented information for the true understanding of content [13]. Previous work has demonstrated the idea of a “beginner’s bubble” [49]. Learners are initially cautious and lack comfort in what they know before education. Small increases in learning may result in large leaps of confidence, which may not accurately align with actual knowledge increase [49], likely explaining the control group’s negative correlation. Continued education and challenging and refining of knowledge lead to a leveling of confidence with incremental performance improvement. Girz et al. confirmed this notion by demonstrating that, with intense training, increased comfort in identifying ED symptoms and diagnosing and managing EDs does relate more accurately to increased knowledge [45].

Patients with EDs present to their healthcare providers significantly more frequently than their peers five years before the ED diagnosis [50]. Patients present to their provider for gynecological, gastrointestinal, and psychological complaints, symptoms directly related to an ED, but the diagnosis is often missed. Even in cases where the diagnosis is identified, an alarming number of providers fail to provide appropriate treatment referrals [51]. While improvement in providers’ self-perceived knowledge and skills demonstrated in this study is promising, these results may not truly reflect actual practice and management change, requiring further research.

While most participants were satisfied with their learning experience, more participants in the control group reported being satisfied with their learning experience than the intervention group. Despite this difference in satisfaction, the intervention group performed better. Previous literature supports that learning style preference does not necessarily correlate with improved grades or course scores [52]. Additionally, student satisfaction with an educational method does not correlate with greater effectiveness of the technique [53]. The current study measured satisfaction to assess how acceptable retrieval practice was among trainees; however, our results support that satisfaction with a learning method alone should not be used to indicate the effectiveness or value of a learning method.

Limitations of the study

In this study, the intervention group had more higher-level trainees than the control group. This discrepancy may have influenced the overall knowledge base, affecting the knowledge base trainees had to draw upon, and impacting scores on the pre- and post-rotation knowledge tests. This difference could also influence self-perceived comfort in treating patients with EDs. More seasoned residents have more practice incorporating new evidence into illness scripts, which could, in turn, affect the amount of comfort they would express on the post-rotation survey. However, prior studies have refuted this concept, showing that the year of residency did not predict comfort with diagnosing or treating EDs, rather the intensity of training had a far larger impact in predicting comfort with diagnosing and treating EDs [45]. Our study likely reflects this finding since most trainees lacked both training and patient experience before the rotation.

Our study contained a small number of participants and a limited number of clusters. The intraclass correlation for the knowledge test model was 0.17, which indicates that 17% of the variance in the data is explained by the random effect for the block. This is higher than the estimate in the power calculation of 0.01-0.05. The size of clusters was limited due to resident assignment onto the rotation. The number of clusters was limited due to time constraints and the COVID-19 pandemic.

Our data represent only one institution and only test the concept of retrieval practice within the confines of eating disorder education. A multi-center study using retrieval practice in broader contexts may provide better insight into the feasibility of incorporating retrieval practice into existing curricula and make results more generalizable.

Strengths of study

Each participant received unique knowledge tests at each point throughout the rotation (start and finish), thus eliminating the possibility of either the control or the intervention group remembering previous responses. Groups were blinded to intervention and control procedures. Participants were only informed that they were taking part in an educational study to improve their understanding of EDs. Trainees joined the rotation at all points of training. During the block’s first learning session, all trainees participated in a case-based discussion on a theoretical ED patient as an introduction. During this session, participants reviewed diagnosing, working up, and treating EDs so that all participants went into the remaining learning sessions with a similar knowledge base.

## Conclusions

This is the first prospective, cluster-randomized trial evaluating a potential method for educating residents and medical students on ED recognition, diagnosis, and treatment. Recent studies have focused on improving ED education for healthcare professionals who have completed their post-graduate training. Among primary care providers, formal ED curricula and interventions that utilize active learning have the potential to increase providers’ knowledge of ED assessment and treatment significantly and may also improve practice behaviors. Our results show promise in using retrieval practice to educate our trainees while in medical school and residency. Future research should focus on determining if increased knowledge correlates with improvements in medical practice. Studies are needed to determine if improvement in self-reported comfort, confidence, knowledge, and skills results in improved outcomes through earlier recognition, diagnosis, and treatment of patients with EDs.

## Appendices

Pre-rotation attitudes survey [1,2]

Name:

Email address:

Year of Training:

Medical Student Year 3 ____

Medical Student Year 4 ____

Resident PGY-1 ____

Resident PGY-2  ____

Resident PGY-3 _____

Resident PGY-4 _____

Resident PGY-5 _____

What is your current training program:

_____  Medical school. If so, name of Medical school: __________________________________

_____  Residency program. If so, what Residency program and specialty:

______________________________________________________________________________

Preferred specialty upon completion of training:

Age:

Ethnicity:

Gender:

What is your preferred learning style?  Select the top two that apply:

·         Visual (spatial): You prefer using pictures, images and spatial understanding.

·         Aural (auditory-musical): You prefer using sound and music.

·         Verbal (linguistic): You prefer using words, both in speech and writing.

·         Physical (kinesthetic): You prefer using your body, hands and sense of touch

·         Logical (mathematical): You prefer using logic, reasoning and systems

·         Social (interpersonal): You prefer to learn in groups our with other people

·         Solitary (intrapersonal): You prefer to work alone and use self-study

Have you treated patients with eating disorders during your medical/residency training? 

___ yes   ___no

If yes, how many cases have you participated in diagnosis or treatment?

____ 0

____ 1-5

____ 6-10

____ 11-15

____ More than 15

Have you received formal education on eating disorders during your medical/residency training? 

___ yes   ___no

If yes, approximately how many hours of training have you received so far?

____ 0

____ 1-2

____ 3-4

____ 5-6

____ More than 6

How comfortable are you in managing patients with eating disorders?

___ Extremely uncomfortable ___ Somewhat uncomfortable

___ Neither comfortable or uncomfortable

___ Somewhat comfortable

___ Extremely comfortable

How important is it to you to be knowledgeable about eating disorders? 

___ Extremely unimportant ___ Somewhat unimportant

___ Neither important or unimportant

___ Somewhat important

___ Extremely important

How comfortable are you in treating adolescents? 

___ Extremely uncomfortable ___ Somewhat uncomfortable

___ Neither comfortable or uncomfortable

___ Somewhat comfortable

___ Extremely comfortable

Have you ever found out that a patient had a severe eating disorder, but you had missed the signs and diagnosis?

___ yes ___ no

If yes, what do you think prevented you from recognizing the signs and diagnosing the eating disorder? (Check all that apply)

___ Weight was within the normal range

___ Unsure about what questions to ask the patient and their family

___ Patient was not forthcoming or honest with their answers when you did ask about diet, exercise, and/or body image

___ Eating disorder symptoms were not the presenting concern, so you did not think to screen

___ Insufficient training in the skills necessary to adequately screen for an eating disorder

___ Upon questioning, the patient denied having any kind of eating disorder or becoming defensive

Other: _____________________________________________

Please rate your current knowledge regarding each of the following items with the following scale:

1: Very low (i.e. very uninformed)

2: Low

3: Moderate

4: High

5: Very High (i.e. very informed)

·         The prevalence rates of eating disorders in the United States _____

·         The local resources available to patients struggling with an eating disorder_____

·         The current best practices for working with eating disorders_____

·         The other conditions that often co-occur with eating disorders_____

·         The most effective ways to talk about weight management and weigh-ins with patients struggling with eating disorders_____

·         The common presenting complaints of patients struggling with an eating disorder_____

·         The risk factors associated with developing eating disorders_____

·         The indicators of recovery for patients with eating disorders_____

·         The factors contributing to relapse in patients with eating disorders_____

Please rate your current skill level regarding each of the following items with the following scale:

1: Very low (i.e. very uninformed)

2: Low

3: Moderate

4: High

5: Very High (i.e. very informed)

·         Asking a patient about their eating habits, weight, and body image without unduly raising their defenses _____

·         Talking with a patient about their eating disorder _____

·         Making appropriate referrals to eating disorder specialists _____

·         Handling a situation where you believe a patient has an eating disorder, but they are denying it or minimizing the impact on their physical and emotional functioning/well-being _____

·         Handling a patient’s disclosure that they have an eating disorder _____

·         Conducting an eating disorder screening _____

Rate how strongly you agree or disagree with the following statements.

Universal screening for eating disorders is appropriate even when an eating disorder or weight is not the presenting concern. 

___Strongly Agree 

___Mildly Agree 

___Neither Agree nor disagree 

___Mildly Disagree 

___Strongly Disagree 

Do you feel well-trained to recognize eating disorders? 

___Strongly Agree 

___Mildly Agree 

___Neither Agree nor disagree 

___Mildly Disagree 

___Strongly Disagree 

Do you feel well-trained to diagnose eating disorders? 

___Strongly Agree 

___Mildly Agree 

___Neither Agree nor disagree 

___Mildly Disagree 

___Strongly Disagree 

Do you feel well-trained to treat eating disorders?

___Strongly Agree 

___Mildly Agree 

___Neither Agree nor disagree 

___Mildly Disagree 

___Strongly Disagree 

This condition is psychological rather than medical.

___Strongly Agree 

___Mildly Agree 

___Neither Agree nor disagree 

___Mildly Disagree 

___Strongly Disagree 

Symptoms of this illness are fairly common and will resolve over time, without specific treatment.

___Strongly Agree 

___Mildly Agree 

___Neither Agree nor disagree 

___Mildly Disagree 

___Strongly Disagree 

This illness is a “severe and enduring” mental illness.

___Strongly Agree 

___Mildly Agree 

___Neither Agree nor disagree 

___Mildly Disagree 

___Strongly Disagree 

Patients with this illness are largely responsible for his/her own condition.

___Strongly Agree 

___Mildly Agree 

___Neither Agree nor disagree 

___Mildly Disagree 

___Strongly Disagree 

This illness has major consequences on a patient’s quality of life.

___Strongly Agree 

___Mildly Agree 

___Neither Agree nor disagree 

___Mildly Disagree 

___Strongly Disagree 

This condition causes difficulties for a patient’s family and friends.

___Strongly Agree 

___Mildly Agree 

___Neither Agree nor disagree 

___Mildly Disagree 

___Strongly Disagree 

This condition is likely to be chronic.

___Strongly Agree 

___Mildly Agree 

___Neither Agree nor disagree 

___Mildly Disagree 

___Strongly Disagree 

Treatment is highly effective for patients with these symptoms.

___Strongly Agree 

___Mildly Agree 

___Neither Agree nor disagree 

___Mildly Disagree 

___Strongly Disagree 

Patients can do a lot to control these symptoms.

___Strongly Agree 

___Mildly Agree 

___Neither Agree nor disagree 

___Mildly Disagree 

___Strongly Disagree 

I enjoy working with eating disorder patients and their families.

___Strongly Agree 

___Mildly Agree 

___Neither Agree nor disagree 

___Mildly Disagree 

___Strongly Disagree 

The author adapted the survey from Currin et al. [11] and Linville et al. [12].

Post-rotation attitude survey (intervention) [1,2]

Name:

Preferred specialty upon completion of training:

Have you treated patients with eating disorders during this rotation? 

___ yes   ___no

If yes, how many cases have you participated in diagnosis or treatment?

____ 0

____ 1-5

____ 6-10

____ 11-15

____ More than 15

How comfortable are you in managing patients with eating disorders?

___ Extremely uncomfortable ___ Somewhat uncomfortable

___ Neither comfortable or uncomfortable

___ Somewhat comfortable

___ Extremely comfortable

How important is it to you to be knowledgeable about eating disorders? 

___ Extremely unimportant ___ Somewhat unimportant

___ Neither important or unimportant

___ Somewhat important

___ Extremely important

How comfortable are you in treating adolescents? 

___ Extremely uncomfortable ___ Somewhat uncomfortable

___ Neither comfortable or uncomfortable

___ Somewhat comfortable

___ Extremely comfortable

Have you ever found out that a patient had a severe eating disorder, but you had missed the signs and diagnosis?

___ yes ___ no

If yes, what do you think prevented you from recognizing the signs and diagnosing the eating disorder? (Check all that apply)

___ Weight was within the normal range

___ Unsure about what questions to ask the patient and their family

___ Patient was not forthcoming or honest with their answers when you did ask about diet, exercise, and/or body image

___ Eating disorder symptoms were not the presenting concern, so you did not think to screen

___ Insufficient training in the skills necessary to adequately screen for an eating disorder

___ Upon questioning, the patient denied having any kind of eating disorder or becoming defensive

Other: _____________________________________________

Please rate your current knowledge regarding each of the following items with the following scale:

1: Very low (i.e. very uninformed)

2: Low

3: Moderate

4: High

5: Very High (i.e. very informed)

·         The prevalence rates of eating disorders in the United States _____

·         The local resources available to patients struggling with an eating disorder_____

·         The current best practices for working with eating disorders_____

·         The other conditions that often co-occur with eating disorders_____

·         The most effective ways to talk about weight management and weigh-ins with patients struggling with eating disorders_____

·         The common presenting complaints of patients struggling with an eating disorder_____

·         The risk factors associated with developing eating disorders_____

·         The indicators of recovery for patients with eating disorders_____

·         The factors contributing to relapse in patients with eating disorders_____

Please rate your current skill level regarding each of the following items with the following scale:

1: Very low (i.e. very uninformed)

2: Low

3: Moderate

4: High

5: Very High (i.e. very informed)

·         Asking a patient about their eating habits, weight, and body image without unduly raising their defenses _____

·         Talking with a patient about their eating disorder _____

·         Making appropriate referrals to eating disorder specialists _____

·         Handling a situation where you believe a patient has an eating disorder, but they are denying it or minimizing the impact on their physical and emotional functioning/well-being _____

·         Handling a patient’s disclosure that they have an eating disorder _____

·         Conducting an eating disorder screening _____

Rate how strongly you agree or disagree with the following statements.

Universal screening for eating disorders is appropriate even when an eating disorder or weight is not the presenting concern. 

___Strongly Agree 

___Mildly Agree 

___Neither Agree nor disagree 

___Mildly Disagree 

___Strongly Disagree 

Do you feel well-trained to recognize eating disorders? 

___Strongly Agree 

___Mildly Agree 

___Neither Agree nor disagree 

___Mildly Disagree 

___Strongly Disagree 

Do you feel well-trained to diagnose eating disorders? 

___Strongly Agree 

___Mildly Agree 

___Neither Agree nor disagree 

___Mildly Disagree 

___Strongly Disagree 

Do you feel well-trained to treat eating disorders?

___Strongly Agree 

___Mildly Agree 

___Neither Agree nor disagree 

___Mildly Disagree 

___Strongly Disagree 

This condition is psychological rather than medical.

___Strongly Agree 

___Mildly Agree 

___Neither Agree nor disagree 

___Mildly Disagree 

___Strongly Disagree 

Symptoms of this illness are fairly common and will resolve over time, without specific treatment.

___Strongly Agree 

___Mildly Agree 

___Neither Agree nor disagree 

___Mildly Disagree 

___Strongly Disagree 

This illness is a “severe and enduring” mental illness.

___Strongly Agree 

___Mildly Agree 

___Neither Agree nor disagree 

___Mildly Disagree 

___Strongly Disagree 

Patients with this illness are largely responsible for his/her own condition.

___Strongly Agree 

___Mildly Agree 

___Neither Agree nor disagree 

___Mildly Disagree 

___Strongly Disagree 

This illness has major consequences on a patient’s quality of life.

___Strongly Agree 

___Mildly Agree 

___Neither Agree nor disagree 

___Mildly Disagree 

___Strongly Disagree 

This condition causes difficulties for a patient’s family and friends.

___Strongly Agree 

___Mildly Agree 

___Neither Agree nor disagree 

___Mildly Disagree 

___Strongly Disagree 

This condition is likely to be chronic.

___Strongly Agree 

___Mildly Agree 

___Neither Agree nor disagree 

___Mildly Disagree 

___Strongly Disagree 

Treatment is highly effective for patients with these symptoms.

___Strongly Agree 

___Mildly Agree 

___Neither Agree nor disagree 

___Mildly Disagree 

___Strongly Disagree 

Patients can do a lot to control these symptoms.

___Strongly Agree 

___Mildly Agree 

___Neither Agree nor disagree 

___Mildly Disagree 

___Strongly Disagree 

I enjoy working with eating disorder patients and their families.

___Strongly Agree 

___Mildly Agree 

___Neither Agree nor disagree 

___Mildly Disagree 

___Strongly Disagree 

How satisfied were you with intermittent quizzes as a learning technique?

___Extremely unsatisfied

___Somewhat unsatisfied

___Neither satisfied or unsatisfied

___ Somewhat satisfied

___ Extremely satisfied

If so, what part did you find most conducive to learning?

How much do you feel you are likely to retain knowledge attained with this method of learning? 

___Extremely unlikely

___Somewhat unlikely

___Neither likely or unlikely

___ Somewhat likely

___ Extremely likely

How often have you used the quizzing technique in the past as a learning/study tool?

___Never

___Not often

___Somewhat often

___Often

___I always use this method for learning

How likely are you to use the quizzing technique in the future as a learning or study tool?

___Extremely unlikely

___Somewhat unlikely

___Neither likely or unlikely

___ Somewhat likely

___ Extremely likely

The author adapted the survey from Currin et al. [11] and Linville et al. [12].

Post-rotation attitude survey (standard) [1,2]

Name:

Preferred specialty upon completion of training:

Have you treated patients with eating disorders during this rotation? 

___ yes   ___no

If yes, how many cases have you participated in diagnosis or treatment?

____ 0

____ 1-5

____ 6-10

____ 11-15

____ More than 15

How comfortable are you in managing patients with eating disorders?

___ Extremely uncomfortable ___ Somewhat uncomfortable

___ Neither comfortable or uncomfortable

___ Somewhat comfortable

___ Extremely comfortable

How important is it to you to be knowledgeable about eating disorders? 

___ Extremely unimportant ___ Somewhat unimportant

___ Neither important or unimportant

___ Somewhat important

___ Extremely important

How comfortable are you in treating adolescents? 

___ Extremely uncomfortable ___ Somewhat uncomfortable

___ Neither comfortable or uncomfortable

___ Somewhat comfortable

___ Extremely comfortable

Have you ever found out that a patient had a severe eating disorder, but you had missed the signs and diagnosis?

___ yes ___ no

If yes, what do you think prevented you from recognizing the signs and diagnosing the eating disorder? (Check all that apply)

___ Weight was within the normal range

___ Unsure about what questions to ask the patient and their family

___ Patient was not forthcoming or honest with their answers when you did ask about diet, exercise, and/or body image

___ Eating disorder symptoms were not the presenting concern, so you did not think to screen

___ Insufficient training in the skills necessary to adequately screen for an eating disorder

___ Upon questioning, the patient denied having any kind of eating disorder or becoming defensive

Other: _____________________________________________

Please rate your current knowledge regarding each of the following items with the following scale:

1: Very low (i.e. very uninformed)

2: Low

3: Moderate

4: High

5: Very High (i.e. very informed)

·         The prevalence rates of eating disorders in the United States _____

·         The local resources available to patients struggling with an eating disorder_____

·         The current best practices for working with eating disorders_____

·         The other conditions that often co-occur with eating disorders_____

·         The most effective ways to talk about weight management and weigh-ins with patients struggling with eating disorders_____

·         The common presenting complaints of patients struggling with an eating disorder_____

·         The risk factors associated with developing eating disorders_____

·         The indicators of recovery for patients with eating disorders_____

·         The factors contributing to relapse in patients with eating disorders_____

Please rate your current skill level regarding each of the following items with the following scale:

1: Very low (i.e. very uninformed)

2: Low

3: Moderate

4: High

5: Very High (i.e. very informed)

·         Asking a patient about their eating habits, weight, and body image without unduly raising their defenses _____

·         Talking with a patient about their eating disorder _____

·         Making appropriate referrals to eating disorder specialists _____

·         Handling a situation where you believe a patient has an eating disorder, but they are denying it or minimizing the impact on their physical and emotional functioning/well-being _____

·         Handling a patient’s disclosure that they have an eating disorder _____

·         Conducting an eating disorder screening _____

Rate how strongly you agree or disagree with the following statements.

Universal screening for eating disorders is appropriate even when an eating disorder or weight is not the presenting concern. 

___Strongly Agree 

___Mildly Agree 

___Neither Agree nor disagree 

___Mildly Disagree 

___Strongly Disagree 

Do you feel well-trained to recognize eating disorders? 

___Strongly Agree 

___Mildly Agree 

___Neither Agree nor disagree 

___Mildly Disagree 

___Strongly Disagree 

Do you feel well-trained to diagnose eating disorders? 

___Strongly Agree 

___Mildly Agree 

___Neither Agree nor disagree 

___Mildly Disagree 

___Strongly Disagree 

Do you feel well-trained to treat eating disorders?

___Strongly Agree 

___Mildly Agree 

___Neither Agree nor disagree 

___Mildly Disagree 

___Strongly Disagree 

This condition is psychological rather than medical.

___Strongly Agree 

___Mildly Agree 

___Neither Agree nor disagree 

___Mildly Disagree 

___Strongly Disagree 

Symptoms of this illness are fairly common and will resolve over time, without specific treatment.

___Strongly Agree 

___Mildly Agree 

___Neither Agree nor disagree 

___Mildly Disagree 

___Strongly Disagree 

This illness is a “severe and enduring” mental illness.

___Strongly Agree 

___Mildly Agree 

___Neither Agree nor disagree 

___Mildly Disagree 

___Strongly Disagree 

Patients with this illness are largely responsible for his/her own condition.

___Strongly Agree 

___Mildly Agree 

___Neither Agree nor disagree 

___Mildly Disagree 

___Strongly Disagree 

This illness has major consequences on a patient’s quality of life.

___Strongly Agree 

___Mildly Agree 

___Neither Agree nor disagree 

___Mildly Disagree 

___Strongly Disagree 

This condition causes difficulties for a patient’s family and friends.

___Strongly Agree 

___Mildly Agree 

___Neither Agree nor disagree 

___Mildly Disagree 

___Strongly Disagree 

This condition is likely to be chronic.

___Strongly Agree 

___Mildly Agree 

___Neither Agree nor disagree 

___Mildly Disagree 

___Strongly Disagree 

Treatment is highly effective for patients with these symptoms.

___Strongly Agree 

___Mildly Agree 

___Neither Agree nor disagree 

___Mildly Disagree 

___Strongly Disagree 

Patients can do a lot to control these symptoms.

___Strongly Agree 

___Mildly Agree 

___Neither Agree nor disagree 

___Mildly Disagree 

___Strongly Disagree 

I enjoy working with eating disorder patients and their families.

___Strongly Agree 

___Mildly Agree 

___Neither Agree nor disagree 

___Mildly Disagree 

___Strongly Disagree 

How satisfied were you with lectures as a learning technique?

___Extremely unsatisfied

___Somewhat unsatisfied

___Neither satisfied or unsatisfied

___ Somewhat satisfied

___ Extremely satisfied

If so, what part did you find most conducive to learning?

How much do you feel you are likely to retain knowledge attained with this method of learning? 

___Extremely unlikely

___Somewhat unlikely

___Neither likely or unlikely

___ Somewhat likely

___ Extremely likely

How often have you used lectures in the past as a learning/study tool?

___Never

___Not often

___Somewhat often

___Often

___I always use this method for learning

How likely are you to use lectures in the future as a learning or study tool?

___Extremely unlikely

___Somewhat unlikely

___Neither likely or unlikely

___ Somewhat likely

___ Extremely likely

The author adapted the survey from Currin et al. [11] and Linville et al. [12].

Knowledge test - All question version ABC w/ answers

Objectives:

Understand the differences between DSM-5 and prior diagnostic criteria for eating disorders.

Recognize clinical presentations characteristic of anorexia nervosa, bulimia nervosa, and binge-eating disorder.

1A. You are seeing a 14-year-old female in your office for a yearly well-check.  She is a dancer in a local troupe and practices 6-7 days a week with competitions 2 weeks away.  She has always been small but consistently tracked along the 35th percentile for BMI.  Today, she is below the 5th percentile for BMI. A review of systems is positive for fatigue, constipation, and bloating.  She attributes the weight loss to a “lack of appetite” due to “nerves” over the upcoming competition season.  Alone, she admits to fear of weight gain especially with competitions approaching and desire for more weight loss to be “healthy” and to look like “the other girls”.  You suspect an eating disorder and obtain an EKG in the office. 

Which EKG findings are most likely to occur in patients with Anorexia Nervosa?

A.      Normal EKG with sinus rhythm and/or shortening of the QT interval with Osborn waves (J waves).

B.      Peaked T waves with a narrow base, depressed ST segment, short QT interval and/or prolongation and increased dispersion of the QT interval.

C.      Sinus bradycardia with low-voltage on EKG and/or tall, peaked T waves with a narrow base, depressed ST segment, and short QT interval.

D.     Sinus bradycardia with low-voltage on EKG and prolongation and/or increased dispersion of the QT interval.

1B.  You are seeing a 15-year-old female in your office for a yearly well-check.  She is a gymnast in a local club and practices 2-3 hours a day, 6-7 days a week.  She has always been small but consistently tracked along the 37th percentile for BMI.  Today, she is below the 5th percentile for BMI. A review of systems is positive for hair thinning, constipation, and bloating.  She attributes the weight loss to a “lack of appetite” due to “nerves” over the upcoming qualifying season.  Alone, she admits she’s afraid to gain weight, especially with qualifiers only two weeks away.  She states the “smaller” she is, the better she’ll do on beam and floor routine.  You suspect an eating disorder and obtain an EKG in the office. 

Which EKG finding is least likely to occur in patients with Anorexia Nervosa?

A.      Prolongation of the QT interval with increased dispersion.

B.      Shortened QT interval with narrow-based peaked T waves and depressed ST segment.

C.      Normal EKG with sinus rhythm.

D.     Sinus bradycardia with low-voltage on EKG.

1C.  You are seeing a 13-year-old female in your office for a yearly sports physical.  She has no significant family history.  She is a dancer in the BalletMet and practices 7 days a week.  She has previously tracked along the 35th percentile for BMI.  Today, she is at the 9th percentile. A review of systems is positive for cold intolerance, constipation, and thinning hair.  She attributes the weight loss to “healthy eating” as performances are 2 weeks away and she wanted to “get in shape”.  Alone, she admits to a fear of fat on her body and that she body checks in mirrors when no one is looking.

You suspect an eating disorder.  Due to concerning vitals in a clinic, an EKG is obtained.  Which of the following EKG findings is most likely in this patient?

A.      Normal EKG with a heart rate of 75 BPM.

B.      Sinus bradycardia with a heart rate of 42 BPM and low voltage.

C.      Abnormal EKG with sinus tachycardia and a heart rate of 109 BPM.

D.     Normal EKG with a heart rate of 61 BPM with sinus arrhythmia.

Objective: Plan appropriate management for anorexia nervosa, bulimia nervosa, and binge-eating disorder

2A. You are seeing a 15-year-old male in your clinic for a Well Adolescent Exam.  He has a prior history of being teased at school for being “chunky”.  He had always tracked close to the 88th percentile for BMI.  The family has recently returned from a vacation in California, where they saw him in a swimsuit and noticed weight loss.  Today his BMI is at the 50th percentile.  Mom and dad state he’s “restricting” and will only eat potato chips and pretzels. He always complains of being tired.  They’ve also noticed he complains of dizziness more often.  In private, he tells you he has decreased morning erections.  On exam, he appears tan and has symptomatic orthostatic hypotension with dizziness.  Otherwise, the exam is unremarkable.

Which set of lab testing is most likely to lead to diagnosis?

A.      Total testosterone, free testosterone and sex hormone binding globulin.

B.      Electrolytes, calcium, magnesium, phosphorus and prealbumin.

C.      TSH, Free T4 and thyroid peroxidase antibody.

D.     Chem 10, serum cortisol, morning plasma ACTH.

2B. You are seeing a 14-year-old male in your clinic for a well-child check.  He has frequently tracked along the 92nd percentile for BMI.  Today he comes in, and over the past year has dropped to the 50th percentile for BMI.  Mom and dad praise him for all the “hard work” he has put into losing weight.  He has joined the baseball team and even runs every morning before school, which parents say speaks to his “dedication” and “work ethic”.

What constellation of symptoms would make you most concerned about an eating disorder in this patient?

A.      Fatigue, elevated blood pressure, loss of appetite, malaise, changes in urination

B.      Increased muscle growth, hyperactivity, disrupted sleep patterns, increased acne

C.      Abdominal pain, chronic diarrhea, fatigue, behavioral issues, vomiting

D.     Fatigue, constipation, loss of morning erections and bradycardia

2C. You are seeing a 16-year-old male in your clinic for a well-child check.  His family describes him as being “chubby” previously.  He has always tracked close to the 90th percentile for BMI. The family became concerned while on vacation in Florida, where they saw him in swim trunks and noticed weight loss.  Today his BMI is at the 45th percentile.  Mom and dad state he’s “not eating anymore,” preferring only to eat seasalt chips and pretzels. He always complains of being tired.  They’ve also noticed he complains of dizziness.  In private, he tells you he has decreased morning erections.  On exam, he is tired appearing, has tan skin, a flat affect, and has symptomatic orthostatic hypotension on vitals with reported dizziness.

Which set of lab testing is most likely to lead to diagnosis?

A.      Blood urea nitrogen, serum creatinine, and urinalysis.

B.      Electrolytes, magnesium, phosphorus, and prealbumin.

C.      Total testosterone, free testosterone, and sex hormone-binding globulin.

D.     Chem 10, serum cortisol, and morning plasma ACTH.

Objective: Recognize clinical presentations characteristic of anorexia nervosa, bulimia nervosa, and binge-eating disorder.

3A. A 15-year-old female is taken to the emergency department after a finding of bradycardia to 52 BPM was discovered by her pediatrician.  She has a history of weight loss of 30 pounds over the last 4-5 months and mom is certain she has heard her daughter vomit after meals quite frequently.  She’s made comments about wishing to be “thinner”.  In the emergency room, she is defensive.  Vitals show bradycardia and orthostatic hypotension with reported dizziness.

Which of the following EKG findings is most likely in this situation?

A.      Sinus tachycardia, short PR interval, and diffuse ST depressions.

B.      Shortening of the QT interval with Osborn waves (J waves).

C.      Tall, peaked T waves with a narrow base, depressed ST segment, and short QT interval.

D.     Flattened T wave, depressed ST segment, and U wave.

3B. A 13-year-old female is taken to the emergency department after being found lying on the kitchen floor, unresponsive, by her sister.  She has a history of weight loss of 30 pounds over the last 4-5 months and mom is quite certain she secretly exercises in her room after meals.  Mom found Snapchat messages to friends that she wants to be “skinny”.  At the emergency room, she is defensive and states that she “just passed out for a second” due to “dehydration”.

Which of the following EKG findings is most likely in this situation?

A.      Shortening of the QT interval with Osborn waves (J waves).

B.      Sinus tachycardia, short PR interval, and diffuse ST depressions.

C.      Prolongation and increased dispersion of the QT interval.

D.     Tall, peaked T waves with a narrow base, depressed ST segment, and short QT interval.

3C. A 17-year-old female is taken to the Urgent Care with symptoms of weakness and fatigue.  She has a history of weight loss and mom is concerned “something is wrong with her”.  Mom asks about acute flaccid myelitis because she “heard about it on the TV”.  When questioned separately, the patient admits she sometimes “makes herself sick” if she “eats too much”.  An EKG is obtained. 

Which of the following EKG findings is most likely to be found on further evaluation?

A.      Sinus tachycardia, short PR interval, and diffuse ST depressions.

B.      Flattened T wave, depressed ST segment, and U wave.

C.      Shortening of the QT interval with Osborn waves (J waves).

D.     Tall, peaked T waves with a narrow base, depressed ST segment, and short QT interval.

Objective:  Understand the differences between DSM-5 and prior diagnostic criteria for eating disorders.

4A. A 14-year-old girl presents to your office with her mother with a chief complaint of “fatigue”.  Mom also feels her daughter is getting “thin” and is concerned that her daughter could have an “overactive thyroid” because the patient’s great aunt also had thyroid issues.  Upon further examination, it is found that her BMI has decreased from the 75th to the 23rd percentile in the last 6 months, to which the patient states “I haven’t had an appetite, I can only get breakfast down”.  You complete a physical exam.  Pertinent findings include thin appearance, heart rate of 49 BPM supine, and dry skin.  You order labs, including a Chem 10, LFTs, CBC, TSH, Free T4, amylase, vitamin D and an EKG. 

What additional piece of information would lead to the diagnosis of Anorexia Nervosa in this patient?

A.      She has experienced increased anxiety and irritability, changes in her menstrual pattern, changes in bowel patterns, and fine, brittle hair that breaks easily.

B.      She engages in recurrent episodes of out-of-control eating involving an amount of food that is definitely larger than what most individuals would eat in a similar period of time.

C.      Patient confides in private that she’s afraid of seeing fat on her body.

D.     Food avoidance is influenced by food texture and a fear of choking.

4B. A 16-year-old girl presents to your office with her grandmother due to weight loss.  Grandmother feels her granddaughter is getting “thin” and is concerned that she could have “gluten intolerance” because she saw on tv this could cause “abdominal issues”.  Upon further examination, it is found that the patient’s weight has decreased from 61.2 kg to 48 kg in the last 6 months, to which the patient states “I haven’t had an appetite, my stomach hurts whenever I eat anything”.  You complete a physical exam.  Pertinent findings include a supine heart rate of 55 BPM, thin appearance, and thinning hair.  You order labs, including a Chem 10, LFTs, CBC, TSH, Free T4, amylase, vitamin D. 

What additional piece of information would confirm your suspicion of anorexia nervosa, restricting type in this patient?

A.      History and lab findings confirming a significant nutritional deficiency.

B.      Menses was at 12 years old, but has not had a menstrual period in greater than three consecutive months.

C.      Patient voices fear of weight gain.

D.     Labs reveal a K of 2.8 and an elevated amylase.

4C. A 13-year-old girl presents to your office with her mother with a chief complaint of “fatigue”.  Mom states her daughter is “too skinny” and is concerned that she could have “thyroid issues” because the patient’s cousin had an “overactive thyroid gland”.  Upon further examination, it is found that her BMI has decreased from the 75th to the 25th percentile in the last 6 months, to which the patient states “I’m just not hungry.”  You complete a physical exam.  Pertinent findings include a supine heart rate of 60 BPM, thin appearance, and lanugo.  You order labs, including a Chem 10, LFTs, CBC, TSH, Free T4, amylase and vitamin D.

What additional piece of information would support a diagnosis of Anorexia Nervosa in this patient?

A.      Physical exam and lab findings confirming significant nutritional deficiency.

B.      She has not had a period in greater than three months consecutively.

C.      Dependence on enteral feeding or oral nutritional supplements.

*D.     Patient hyper-exercises despite having already lost an unhealthy amount of weight*.

Objectives:

Understand the differences between DSM-5 and prior diagnostic criteria for eating disorders.

Distinguish avoidant/restrictive food intake disorder from other eating disorders.

5A. A father brings his 15-year-old son to your clinic with complaints that his son has a 5-month history of “feeling tired” and “losing weight”. He’s concerned that “something is wrong with him” because he has lost 19 lbs. after an episode of vomiting after eating “bad potato salad” at a family picnic 5 months ago. He is active in basketball and currently goes to practice 5 times a week for about 2 hours each practice.  He states he just “hasn’t had an appetite” but that he wants to “gain some muscle” before the season starts.  On review of systems, he complains of constipation and loss of morning erections. He denies recent vomiting. Vital signs reveal heart rate of 54 beats per minute, respiratory rate of 14 breaths per minute, and blood pressure of 94/54 mmHg. The father raises the issue of an eating disorder.

Which of the following eating disorders is the most likely diagnosis for this patient?

A.      Anorexia nervosa, restricting type.

B.      Avoidant/restrictive food intake disorder.

C.   Atypical anorexia nervosa.

D.   Eating Disorder Not Otherwise Specified.

5B. A father brings his 14-year-old daughter to your office with complaints that his daughter has a 7-month history of fatigue and weight loss. He’s concerned that she has lost 19 lbs after an episode of vomiting after eating “bad chicken salad” at a family picnic 8 months ago. She is active in sports and currently goes to volleyball practice 5 times a week for about 1-2 hours each practice.  His daughter denies fear of becoming fat and denies purposefully cutting down on meals, stating she just “doesn’t feel hungry” and doesn’t want to “be sick”. On review of systems, she complains of constipation and she has not had a menstrual period for 4 months. Menarche was at age 11 years. She denies recent vomiting. Vital signs reveal heart rate of 51 beats per minute, respiratory rate of 12 breaths per minute, and blood pressure of 102/56 mmHg. The father raises the issue of an eating disorder.

Which of the following eating disorders is the most likely diagnosis for this patient?

A.      Eating Disorder Not Otherwise Specified.

B.      Anorexia nervosa, restricting type.

C.      Avoidant/restrictive food intake disorder.

D.     Atypical anorexia nervosa.

5C. A father brings his 14-year-old son to your office with complaints that his son has a 6-month history of weight loss and irritability. His son has lost 26 lbs. over the last 6 months. He is active in sports and currently goes to football practice 4 times a week with scrimmages on the weekend.  He also runs 4 miles on days he doesn’t have practice.  He admits he is scared of becoming fat and only eats before practices. On review of systems, he complains of constipation and fatigue.  He denies vomiting. Vital signs reveal a heart rate of 49 beats per minute, respiratory rate of 14 breaths per minute, and blood pressure of 85/40mmHg. The father raises the issue of an eating disorder.

Which of the following eating disorders is the most likely diagnosis for this patient?

A.      Atypical anorexia nervosa.

B.      Eating Disorder Not Otherwise Specified.

C.      Anorexia nervosa, restricting type.

D.     Avoidant/restrictive food intake disorder.

Objective: Understand the differences between DSM-5 and prior diagnostic criteria for eating disorders.

6A. A 17-year-old girl presents for a routine well check before starting college this fall.  She reports that she has received a full scholarship for academics and soccer.  She is dressed in layers and is noted to weigh 105 lbs.  During a comprehensive history and review of systems, she becomes immediately defensive when questioned about her diet.  Mom states she became vegan about 5 months ago.  You suspect an eating disorder.

Which of the following is included in the DSM-5 diagnostic criteria for anorexia nervosa?

A.      Absence of period for 3 or more months in a patient who previously achieved menarche.

B.      Weight less than 80% of expected body weight.

C.      Disturbance in the way one’s body weight or shape is experienced.

D.     Presence of bradycardia or other serious physical manifestations of malnutrition.

6B. A 16-year-old girl presents for routine sports physicals before starting soccer.  She is dressed in layers and is noted to be at the 8th percentile for BMI.  During a comprehensive history and review of systems, she becomes immediately defensive when questioned about her diet.  Dad states she became a pescatarian about 6 months ago.  You suspect an eating disorder.

Which of the following is included in the DSM-5 diagnostic criteria for anorexia nervosa?

A.      Persistent exercise twice a day even though current weight is significantly low.

B.      Weight less than 85% of expected body weight.

C.      Heart rate <55 bpm.

D.     Persistent absence of a period for 3 or more consecutive months.

6C. A 17-year-old girl presents for a routine well-check before starting college this fall.  She reports that she has received a full scholarship for academics and basketball.  She is dressed in baggy clothes and is noted to have dropped from the 45th percentile for BMI to the 6th percentile for BMI.  During a comprehensive history and review of systems, you suspect an eating disorder.

Which of the following is included in the DSM-5 diagnostic criteria for anorexia nervosa?

A.      BMI below the 30th percentile for adolescents.

B.      Evidence of nutritional deficiency on the exam.

C.      Persistent restriction of energy intake that leads to significantly low body weight.

D.     Absence of the period for 3 or more months in a patient who previously achieved menarche.

Objectives:

Describe the multidisciplinary nature of treatment teams

Describe the goals of treatment for eating disorder patients

Describe basic levels of care (medical hospitalization, residential treatment, and levels of outpatient treatments)

7A. Which of the following is false of treatment and recovery of eating disorders? 

A.      Use of medications such as fluoxetine or topiramate may reduce binge eating and purging in patients with bulimia nervosa.

B.      Weight gain in the first year is predictive of success in restrictive eating disorders.

C.      With binge-eating disorder it’s important to institute a weight management plan.

D.     Being obese, younger, and having a chronic illness are risk factors for delayed diagnosis or anorexia nervosa.

7B. Which of the following is false of treatment and recovery of eating disorders? 

A.      Being young, obese, or having a chronic illness are risk factors for delayed diagnosis of anorexia nervosa.

B.      With binge-eating disorder it’s important to institute a weight management plan.

C.      Higher BMI at the transition to outpatient care is predictive of relapse in anorexia nervosa.

D.     Weight gain in the first month is predictive of success in restrictive eating disorders.

7C. Which of the following is true of treatment and recovery of anorexia nervosa?

A.      Treatment outcomes are generally superior in the inpatient setting.

B.      Failure to gain weight in the first month of illness for patients with restrictive eating disorders corresponds with a lower likelihood of recovery at one year.

C.      Certain psychotherapies such as family-based treatment and cognitive behavioral therapy are ineffective when treating anorexia nervosa in adolescents.

D.     There is clear evidence to support the use of anti-depressants to promote weight gain in adolescent patients with anorexia nervosa (Grade B).

Objectives:  Recommend appropriate management of eating disorders based on

v  Criteria necessitating inpatient medical admission for a patient with anorexia nervosa

v  Presence or absence of medical manifestations of refeeding syndrome in an adolescent with severe malnutrition

8A. A 16-year-old female presents with a significant history of weight loss (50 pounds over the past 6 months), excessive exercise, and restricted intake of food.  Her last menstrual period was 4 months ago.  She denies self-induced vomiting or diuretic use.

Upon additional evaluation, which of the following findings is considered criteria for hospital admission in this patient?

A.      74% of ideal body weight.

B.      Blood pressure 90/52 mmHg.

C.      Heart rate 55 bpm.

D.     Temperature 97.0F (36.1C).

8B. A 15-year-old male presents with a significant history of weight loss (50 pounds over the past 6 months), excessive exercise, and restricted intake of food.  He has a loss of morning erections.  He denies self-induced vomiting or diuretic use.

Upon additional evaluation, which of the following findings is considered an indication for hospitalization in this patient?

A.      85% of ideal body weight

B.      Blood pressure 78/48 mmHg

C.      Heart rate 55 bpm

D.     Temperature 97.0F (36.1C)

8C. 4. A 17-year-old female presents with a significant history of weight loss, falling from the 35th percentile for BMI to the 9th percentile.  She participates in excessive exercise, and restricted intake of food.  Her last menstrual period was 6  months ago.  She denies self-induced vomiting or diuretic use.

Upon additional evaluation, which of the following findings is considered criteria for hospital admission in this patient?

A.      Blood pressure 90/52 mmHg.

B.      Heart rate 45 bpm.

C.      77% of ideal body weight.

D.     Temperature 96.5F (35.8C).

Objectives: 

Recommend appropriate management of eating disorders based on:

- Criteria necessitating inpatient medical admission for a patient with anorexia nervosa

- Presence or absence of medical manifestations of refeeding syndrome in an adolescent with severe malnutrition

9A. You are called to consult on a 13-year-old female for acute onset of weakness and paresthesia, admitted to the youth crisis stabilization unit for suicidal ideation.  The patient was recently diagnosed with anorexia nervosa and has lost 25 kg in the last 4 months.  The intern who admitted the patient the first night started the patient on a diet of 3000 kcal/day.  She is currently on day 3 of hospitalization.

Physical exam: 

Cachectic, bilateral lower extremity edema noted

Vitals: Temp 98.2F (36.8C); HR 45; BP 92/54; RR 22; O2 sat 98% on RA

Labs:

Sodium 137 meq/L (normal 135 - 145 meq/L)

Potassium 2.9 mmol/L (normal 3.7 - 5.6 mmol/L)

Chloride 102 meq/L (normal 95 - 106 mmol/L)

BUN 14 mg/dL (normal 5-18 mg/dL)

Cr 0.74 mg/dL (normal 0.40 - 1.10 mg/dL)

Glucose 122 mg/dL (normal 60-115 mg/dL)

Ca 9.4 mg/dL (normal 8 - 10.5 mg/dL)

Mg 1.2 mg/dL (normal 1.5 - 2.4 mg/dL)

Phos 2.4 mg/dL (normal 3.3 - 5.4 mg/dL)

What is the most appropriate next step in management of this patient?

A.      Immediate EKG, make patient NPO, initiate maintenance IV fluids with D5 0.45NS 20K.

B.      Immediate EKG, reduce nutritional support, aggressively correct electrolyte imbalances.

C.      Immediate EKG, reduce nutritional support, initiate maintenance IV fluids with D5 0.45NS 20K, stat repeat chem 7 panel.

D.     Immediate EKG, make patient NPO, start maintenance IV fluids with 0.9 normal saline, aggressively correct electrolyte imbalances.

9B. You are called to consult on a 15-year-old female admitted to the youth crisis stabilization unit for suicidal ideation.  She is complaining of acute onset of chest pain.  The patient was recently diagnosed with anorexia nervosa and has lost 45 kg in the last 7 months.  The intern who admitted the patient the first night started the patient on a diet of 3000 kcal/day.  She is currently on day 3 of hospitalization.

Physical exam: 

Cachectic, bilateral lower extremity edema noted, increased work of breathing

Vitals: Temp 98.2F (36.8C); HR 45; BP 92/54; RR 22; O2 sat 91% on RA

Labs:

Sodium 137 meq/L (normal 136 - 145 meq/L)

Potassium 2.4 meq/L (normal 3.5 - 5 meq/L)

Chloride 102 meq/L (normal 98 - 107 meq/L)

BUN 14 mg/dL (normal 6-22 mg/dL)

Cr 0.74 mg/dL (normal 0.72 - 1.25 mg/dL)

Glucose 122 mg/dL (normal 70-105 mg/dL)

Mg 1.2 mg/dL (normal 1.6 - 2.9 mg/dL)

Phos 1.0 mg/dL (normal 2.3 - 4.7 mg/dL)

Your most immediate concern in this patient is for which of the following?

A.      Esophageal tear secondary to persistent, purposeful purging.

B.      Myocardial infarction secondary to decreased blood flow to the heart.

C.      Superior Mesenteric Artery Syndrome causing the duodenum to be compressed between the aorta and the superior mesenteric artery secondary to malnutrition.

D.     Heart Failure secondary to Refeeding syndrome.

9C. You are called to consult a 14-year-old female in a psychiatric unit for electrolyte abnormalities.  The patient was recently diagnosed with anorexia nervosa and lost 30 kg in the last 5 months.  The intern taking care of the patient started the patient on a diet of 2700 kcal/day.

Physical exam: thin appearing, no acute distress, lanugo, dry vaginal mucosa

Vitals: Temp 98.2F (36.8C); HR - 45; BP - 92/54; RR - 22; O2 sat - 98% on RA, LMP 4 months prior to presentation

Labs:

Sodium 137 meq/L (normal 135 - 145 meq/L)

Potassium 2.9 mmol/L (normal 3.7 - 5.6 mmol/L)

Chloride 102 meq/L (normal 95 - 106 mmol/L)

BUN 14 mg/dL (normal 5-18 mg/dL)

Cr 0.74 mg/dL (normal 0.40 - 1.10 mg/dL)

Glucose 122 mg/dL (normal 60-115 mg/dL)

Ca 9.4 mg/dL (normal 8 - 10.5 mg/dL)

Mg 1.2 mg/dL (normal 1.5 - 2.4 mg/dL)

Phos 2.4 mg/dL (normal 3.3 - 5.4 mg/dL)

The EKG shows ST segment depression, inverted T waves, and U waves with bradycardia.

The patient’s clinical condition is secondary to?

A.      Anorexia Nervosa, binge eating-purging subtype.

B.      Refeeding syndrome.

C.      Bulimia nervosa.

D.     Diuretic and laxative use prior to hospitalization.
